# Sleep quality, perceived stress, and dry eye disease among university students in Ghana: A cross-sectional study of individual and joint associations

**DOI:** 10.1371/journal.pone.0361053

**Published:** 2026-10-27

**Authors:** Benedict Ayobi, Gwendolyn Otwiwa Attram, Bright Vandyke Okyere, Belinda Aba Osrah, John Kwesi Assan, Samuel Abokyi

**Affiliations:** 1 Discipline of Optometry, College of Health Sciences, University of KwaZulu-Natal, Durban, South Africa; 2 School of Optometry and Vision Science, College of Health and Allied Sciences, University of Cape Coast, Cape Coast, Ghana; 3 College of Optometry, University of Houston, Houston, Texas, United States of America; Sanmenxia Central Hospital, Henan University of Science and Technilogy, CHINA

## Abstract

**Purpose:**

Dry Eye Disease (DED) is highly prevalent among university students and may be influenced by modifiable factors such as psychological stress and sleep disturbance. The primary objective of this study was to investigate the associations of sleep disturbance and perceived stress with DED, while the secondary objective was to evaluate their potential interaction.

**Methods:**

This analytical cross-sectional study included a random sample of university students aged 18–34 years. Perceived stress was assessed using the Perceived Stress Scale, and sleep quality was evaluated using the Pittsburgh Sleep Quality Index. DED was diagnosed according to TFOS DEWS II criteria using the Ocular Surface Disease Index, tear break-up time, corneal staining, and Schirmer testing. Robust Poisson regression models were used to estimate relative risks (RRs) for DED associated with sleep disturbance and stress. Interaction effects were assessed on both additive and multiplicative scales.

**Results:**

The prevalence of DED was 77.9%. Sleep disturbance was present in 75.9% of participants, while 21.3% reported high perceived stress. In univariate analyses, both sleep disturbance and stress were significantly associated with DED prevalence (sleep disturbance: χ²(1, 403) = 14.57, *p* < 0.0001; stress: χ²(1, 403) = 4.20, *p* = 0.040). After adjustment for sex and the alternate exposure, poor sleep quality (adjusted RR = 1.11, 95% CI: 1.05–1.18; *p* = 0.001) and high perceived stress (adjusted RR = 1.06, 95% CI: 1.01–1.11; *p* = 0.015) remained independently associated with increased DED risk. Participants with both poor sleep quality and high stress exhibited the highest DED risk (RR = 1.18, 95% CI: 1.10–1.27; *p* < 0.0001), although no significant interaction.

**Conclusion:**

Sleep disturbance and perceived stress were independently associated with an increased risk of DED in this student population. However, there was no evidence that stress modified the association between sleep disturbance and DED.

## Introduction

Dry Eye Disease (DED) is a symptomatic eye disease that is common and significantly impacts the quality of life of individuals of all ages [1]. The Tear Film and Ocular Surface Society (TFOS) defines DED as a “multifactorial disease of the ocular surface that is characterized by the loss of homeostasis of the tear film in which tear film instability, ocular surface inflammation and damage, neurosensory abnormalities, and hyperosmolarity play etiological roles.” [2]

The most reported symptom associated with DED is ocular discomfort. Ocular discomfort is a general term patients use to describe eye strain, dryness, redness, a burning sensation, irritation, and transient blurry vision associated with DED [3,4]. DED affects millions of people globally. The prevalence of DED varies across geographical locations. The global prevalence of DED has been reported to range between 5–50%, with the highest prevalence reported in East Asia and Africa [5,6]. A systematic review of studies reporting on DED showed that the prevalence of DED across Asia, Europe, the Middle East, North America, and Africa was 22.76%, 22.70%, 25.93%, and 5.50%, respectively. It is estimated that about 400 million to 3.7 billion people live with DED [7]. In Ghana, the prevalence of DED has been reported to be between 44.30% − 69.30% [8,9].

Various studies have reported that the risk factors of DED include, among others, age, gender, contact lens wear, refractive surgery, smoking, and the presence of systemic autoimmune diseases such as Sjögren syndrome, thyroid diseases, and pregnancy [7,10–13]. One study found increased DED prevalence among people who use computer screens daily, suggesting an association between digital device use and DED [6]. Emerging evidence suggests modifiable behavioral factors, including sleep and stress, might also contribute to DED [14]. A population-based study among adults in South Korea reported that the prevalence of DED increased with reduced hours of sleep [15]. Another study postulated that insufficient sleep contributes to DED by affecting tear secretion and tear film stability [14]. Additionally, stress has been reported to correlate with DED. Increased stress levels are associated with a higher prevalence of DED. In a cross-sectional study of Saudi Arabian adults, Alrajhi et al. reported that participants who were stressed had 6.5-fold higher odds of DED [16,17]. Additionally, in a population of health workers, Hyon et al. reported that participants who were stressed were 1.2 times more likely to have DED [17]. Thus, the role of sleep duration and stress levels on DED has been extensively studied, although separately. To the best of our knowledge, and based on the available literature, the relationship between DED and the combined effects of stress and sleep remains uncharted and underexplored, especially among university students.

Due to increased academic load, university students are likely to experience stress and sleep fewer hours [18]. We postulate that both increased stress levels and insufficient sleep may be associated with an increased risk of DED among university students. Hence, this study, aimed to assess the relationship between DED and the individual and combined effects of stress levels and sleep disturbances among university students.

## Materials and methods

### Study design and ethical approval

An analytical cross-sectional study was conducted to evaluate the relationships among DED, stress levels, and sleep patterns among students at the University of Cape Coast, Ghana. Participant recruitment ran from 29/06/2022–28/10/2022. The University of Cape Coast is the third-largest university in Ghana by student enrolment, with a student population of about 74,000 [19,20]. The study received ethical approval from the Institutional Review Board (IRB) of the University of Cape Coast, under reference number UCC/IRB/R/1/1582. All participants gave written informed consent. The study protocol strictly adhered to the Declaration of Helsinki guidelines for research involving human subjects. This study is reported in accordance with the Strengthening the Reporting of Observational Studies in Epidemiology (STROBE) guidelines for cross-sectional research.

### Participant recruitment, eligibility criteria, and sampling

The study was conducted at the Optometry Clinic within the School of Optometry and Vision Sciences at the University of Cape Coast, which offers comprehensive primary eye care services. The study participants were registered students actively studying full-time on the university campus at the time of the study. Previous research indicated a high prevalence of Dry Eye Disease (DED), affecting approximately half of this youthful population [9]. To enhance the study’s validity and reduce potential confounding factors, exclusion criteria included students over 35 years old, those with a history of contact lens use, refractive surgery, ocular conditions, diabetes, certain medications affecting the tear film, and habitual smokers or alcohol consumers.

The restriction approach was used to control for prolonged screen time as a confounder, given that prolonged screen exposure is a known risk factor for DED [21]. Accordingly, students reporting ≥3 hours of daily digital screen time were excluded. This cut-off reflects evidence that tertiary students typically spend about three hours per day on digital devices for academic purposes [22,23].

Enrolment into the study was selected using systematic random sampling. Students who agreed to participate were taken to the Optometry Clinic for DED assessment. Based on previously reported DED prevalence and findings from our pilot study [9], the sample size required to assess the risk of DED associated with stress and sleep disturbance was calculated. Using G*Power version 3.1.9.7, the sample size for comparing two independent groups was calculated assuming a statistical power of 80%, a two-sided significance level of 5% (95% confidence level), expected DED proportions of 45% and 70% in the two groups, respectively, and an allocation ratio of 1:2 between the groups. These assumptions yielded a minimum required sample of 134 participants, comprising 89 participants in the exposed group and 45 in the unexposed group. The sample size was subsequently increased to 403 participants to provide greater statistical power to assess the potential joint effects (interaction) of the two factors on DED.

### Questionnaire administration

Three different questionnaires were self-administered to each participant. The Ocular Surface Disease Index (OSDI) was used to assess DED symptoms. The Perceived Stress Scale (PSS) was used to assess participants’ stress levels. The Pittsburgh Sleep Quality Index (PSQI) was used to assess sleep quality and sleep disturbance among subjects.

The Ocular Surface Disease Index (OSDI) comprises 12 questions that relate to ocular symptoms, vision-related function, and environmental triggers. Patients rate their responses on a 0–4 scale, with 0 corresponding to “none of the time” and 4 corresponding to “all of the time.” A final score is calculated, ranging from 0 to 100, with scores 0–12 indicating no DED, 13–22 mild dry eye disease, 23–32 moderate dry eye disease, and greater than 33 severe dry eye disease [24,25].

The Perceived Stress Scale (PSS) questionnaire is a 10-item questionnaire designed to evaluate participants’ self-reported stress levels by assessing thoughts and feelings in the previous month. Each question is scored from 0 (never) to 4 (very often), with a total possible score range of 0–40. Scores ranging from 0–13 would be considered low stress, 14–26 as moderate stress, and 27–40 as high stress [26,27].

The PSQI consists of 24 items, each ranging from 0 (best) to 3 (worst), across seven domains. These seven factors can be broadly categorized into sleep efficiency factors (sleep quality, sleep latency, sleep duration, and habitual sleep efficiency) and sleep disturbance factors (sleep disturbance, use of sleep medications, and daytime disturbance). The global PSQI score ranges from 0 to 21, with 0–5 indicating “good” sleep and 6–21 indicating “poor” sleep, based on the average scores of the seven components [28,29]. The original English versions of all three questionnaires used in this study have previously been validated for use in the Ghanaian population; therefore, no translation or linguistic adaptation was required [9,30,31].

### Tear function examination

Upon completing the study questionnaires, participants underwent clinical examinations to evaluate signs of DED. The assessments began with the fluorescein Tear Break-Up Time (TBUT) test to determine tear film stability, followed by corneal staining, and concluded with the non-anesthetized Schirmer test to measure tear volume, following recommended guidelines [32]. Ocular surface evaluations were performed by licensed clinical optometrists who completed pre-study calibration sessions to ensure high inter-observer reliability. To prevent observer bias, clinical examiners remained strictly masked to participant OSDI questionnaire scores. Examinations were conducted in a standardized clinical environment with an ambient room temperature of 24°C and relative humidity kept between 40% and 60%. Furthermore, to control for diurnal variations in tear volume and stability, all objective tear tests (TBUT, corneal staining, and Schirmer testing) were restricted to afternoon hours between midday (12: 00 pm) and late afternoon (5: 00 pm)

To assess TBUT*,* a drop of saline was applied to the tip of a commercially available sterile fluorescein strip (Tianjin Jingming New Technological Development Co., Ltd., China). The wet strip was placed on the lateral bulbar conjunctiva for about 2 seconds. The participants were asked to blink fully to ensure uniform dye distribution across the eye. The tear film was examined under the slit lamp with a cobalt blue filter. The time interval between the last blink and the appearance of the first dark spot on the cornea was recorded as the TBUT. A cutoff value of less than 10s indicates DED [4].

Corneal staining was evaluated with fluorescein dye and quantified using the Oxford grading scale, which ranges from Grade 0 (no staining) to Grade 5 (severe staining). After dye instillation and patient blinking to ensure even distribution across the corneal surface, examination was conducted under a slit lamp with a cobalt blue filter, highlighting fluorescein-stained areas in bright green to identify epithelial disruption. Central staining has been associated with aqueous-deficient dry eye [32].

For the Schirmer test as described elsewhere [33], a strip of sterile Schirmer paper (Tianjin Jingming New Technological Development Co., Ltd, China) was inserted in the lower cul-de-sac of the fornical conjunctiva at the lateral one-third of the eyelid without anesthesia. The strip was removed after five minutes, and the amount of wetting was read and recorded as the millimeters of wetting. All participants were seated with their eyes closed to minimize the impact of eye movements and environmental factors on the test. A cutoff value of < 5 mm/5 minutes indicates aqueous-deficient DED [4]. DED was defined by the TFOS DEWS II criteria, which require the presence of DED symptoms and at least one positive homeostatic diagnostic tear test in at least one eye [2]. Both eyes were evaluated for clinical signs of DED; however, in accordance with TFOS DEW II criteria, if the clinical values differed between both eyes, the most severely affected eye was considered.

### Data analysis

The collected data were coded and analyzed using SPSS version 26 (IBM SPSS, Inc., Chicago, Illinois). Descriptive results were presented through frequencies, percentages, tables, and summary statistics. The relationship between DED and sleep quality or stress levels was initially examined using the Chi-square test. Next, we assessed the individual effects of stress and sleep disturbance on DED risk, followed by stratified analyses by stress level to examine the joint effect of sleep quality on DED risk. Participants were categorized into low-to-moderate and high-stress levels based on their perceived stress, following recommended guidelines [26,27]. Sleep quality was assessed using the global PSQI, with participants classified as having “good” or “poor” sleep behaviour. A composite variable with four categories representing each possible stress/sleep quality combination was created to analyze the joint effects of perceived stress and sleep quality. Multivariable analyses were conducted using a Poisson regression model with robust error variance, with DED as the outcome variable. Predictors included stress levels, sleep quality, and sex as a covariate. Results were presented as Risk Ratios (RR) with 95% confidence intervals (CI). Additionally, the additive and multiplicative interactions of stress and sleep quality on DED risk were analyzed. Product terms of both variables were introduced into the model to assess potential multiplicative effects. The Relative Excess Risk due to Interaction (RERI), Attributable Proportion due to interaction (AP), and Synergy Index (SI) were calculated to quantify additive interactions. The 95% CIs for these indices were computed using the delta method [34].

## Results

### Participant characteristics

A total of 403 university students participated, including 215 females (53.3%). The mean age was 24.15 ± 3.14 years (range: 18–34), with 224 participants (55.6%) aged 21–25 years and 98.3% younger than 30 years.

### Prevalence and associations of dry eye disease

The mean OSDI score was 40.92 ± 26.26. Based on OSDI criteria, 331 participants (82.1%) reported symptomatic dry eye disease (DED). The mean TBUT was 7.08 ± 3.01 seconds, and the mean Schirmer test value was 18.42 ± 9.55 mm/5 min. Using established diagnostic cut-offs, 314 participants (77.9%) demonstrated tear film instability, while 299 showed corneal staining (Table 1). According to TFOS DEWS II criteria (symptoms plus ≥1 positive homeostatic tear test), 314 participants (77.9%) were diagnosed with DED, of whom 222 (55.1%) had evaporative DED.

**Table 1:** Participants’ demography and dry eye disease (DED)-related characteristics.

	Mean (SD)	Classification	Number	(%)
Sex	–	Male	188	46.7
		Female	215	53.3
Age group	–	18-25	278	69.0
		26-35	125	31.0
OSDI	40.92 (±26.26)	Normal	72	17.9
		Mild dry eye	89	22.1
		Moderate dry eye	10	2.5
		Severe dry eye	232	57.5
PSQI	6.47 (±2.05)	Normal	97	24.1
		Significant sleep disturbance	306	75.9
PSS	22.69 (±5.03)	Low-to-mod. stress	317	78.7
		High stress	86	21.3
TBUT	7.08 (± 3.01)	Tear instability	314	77.9
		Tear stability	89	22.1
Type of DED (Schirmer test)	–	Evaporative type	222	55.1
		Aqueous-deficient type	92	22.8
Corneal staining grading	–	Grade 0: Absent	104	25.8
		Grade 1: Minimal	50	12.4
		Grade 2: Mild	94	23.3
		Grade 3: Moderate	96	23.8
		Grade 4: Marked	59	14.6
		Grade 5: Severe	0	0
TFOS DEWS II diagnostic criteria	–	DED	314	77.9
		NO DED	89	22.1

Participants were categorized according to recommended guidelines as follows: Pittsburgh Sleep Quality Index (PSQI): Normal: 0–4 points; Significant sleep disturbance: ≥ 5 points; Perceived Stress Scale (PSS): Normal-to-moderate stress: 0–26 points; High stress: ≥ 27 points

Low-to-moderate stress was reported by 317 participants (78.7%), and high stress by 86 (21.3%) (mean PSS: 22.69 ± 5.03). The mean PSQI score was 6.47 ± 2.05, indicating poor sleep quality. Among participants with DED, 252 (80.3%) reported sleep disturbances and 74 (23.6%) reported high stress, compared with 54 (60.7%) and 12 (13.5%) among those without DED, respectively (Table 1). Chi-square analysis showed significant associations between DED and sleep disturbance (χ²(1,403) = 14.567, *p* < 0.001) and stress level (χ²(1,403) = 4.201, *p* = 0.040).

### Analyses of sleep disturbance and stress on dry eye disease

In the main-effects model (Table 2), using the Robust Poisson regression, sleep disturbance and perceived stress were independently associated with DED after adjustment for sex and the other predictor variable. Participants with significant sleep disturbance had a higher risk of DED than those with normal sleep (adjusted RR = 1.11, 95% CI: 1.05–1.18; *p* = 0.001). Similarly, participants with high perceived stress had a higher risk of DED compared with those reporting normal-to-moderate stress (adjusted RR = 1.06, 95% CI: 1.01–1.11; *p* = 0.015).

**Table 2:** Main effect model assessing whether sleep disturbance and stress are independently associated with DED.

	DED	Normal	RR (95% CI)	*p-value*
***Risk ratios for single effects***				
*Sleep quality*				
Normal sleep	62	35	Reference	
Significant disturbance	252	54	1.11 (1.05, 1.18)	0.001*
				
*Perceived stress levels*				
Normal-to-moderate	240	77	*Reference*	
High stress	74	12	1.06 (1.01, 1.11)	0.015

Adjusted risk ratio (RR) for sex and either stress (when assessing the effect on sleep quality) or sleep quality (when assessing the effect on stress). RRs were by robust Poisson regression. *A p-value of p < 0.05 was considered statistically significant.

A second model incorporating the sleep disturbance × stress interaction was fitted to assess whether the association between sleep disturbance and DED differed according to stress level (Table 3). In this model, significant sleep disturbance remained independently associated with DED (adjusted RR = 1.11, 95% CI: 1.03–1.20; *p* = 0.004). *However, the association between high stress and DED was no longer statistically significant* (adjusted RR = 1.06, 95% CI: 0.93–1.21; *p* = 0.390). The sleep disturbance × stress interaction term was also not statistically significant (RR = 1.00, 95% CI: 0.87–1.15; *p* = 0.996).

**Table 3:** Model assessing the associated joint effects of sleep disturbance and stress on DED risk and interactions.

	DED	Normal	RR (95% CI)	*p-value*
*Sleep quality*				
Normal sleep	62	35	Reference	
Significant disturbance	252	54	1.11 (1.03, 1.20)	0.004*
				
*Perceived stress levels*				
Normal-to-moderate	240	77	*Reference*	
High stress	74	12	1.06 (0.93, 1.21)	0.390
Sleep x Stress Interaction			1.00 (0.87, 1.15)	0.996
				
***Risk ratios for joint effects***				
*Normal sleep and low-to mod stress*	47	28	*Reference*	
*Normal sleep and high stress*	15	7	1.03 (0.91, 1.18)	0.620
*Sleep disturbance and low-to mod stress*	193	49	1.11 (1.03, 1.19)	0.007*
*Sleep disturbance and high stress*	59	5	1.18 (1.10, 1.27)	<0.0001*

Risk ratio (RR) with the inclusion of interaction between sleep and stress. RRs were by robust Poisson regression. *A p-value of p < 0.05 was considered statistically significant.

The joint-effects analysis nevertheless demonstrated a graded pattern in the risk of DED according to combinations of sleep disturbance and perceived stress. Using participants with normal sleep and normal-to-moderate stress as the reference group, those with normal sleep and high stress had a similar risk of DED (RR = 1.03, 95% CI: 0.91–1.18; p = 0.620). Participants with sleep disturbance and normal-to-moderate stress had a significantly higher risk of DED (RR = 1.11, 95% CI: 1.03–1.19; p = 0.007), while those with both sleep disturbance and high stress had the highest relative risk (RR = 1.18, 95% CI: 1.10–1.27; p < 0.0001). The results on interaction on the additive scale showed confidence intervals for RERI and AP include zero and that for the SI included one, providing no evidence of a statistically significant interaction between sleep disturbance and stress (Table 4).

**Table 4:** Additive interaction measures for sleep disturbance and stress in relation to dry eye disease.

Additive Interaction Measure	Estimate	95% Confidence Interval	*p-value*
Relative Excess Risk due to Interaction (RERI)	0.04	−0.16 to 0.17	0.635
Attributable Proportion due to interaction (AP)	0.01	−0.13 to 0.14	0.885
Synergy Index (SI)	1.04	0.42 to 2.60	0.933

### Sensitivity analysis

To assess robustness, analyses were repeated with age included as a covariate. Consistent with the original model, no significant multiplicative interaction (RR: 1.01, 95% CI: 0.88–1.16, *p* = 0.888) or additive interaction (RERI: 0.02, 95% CI: −0.14 to 0.18, *p* = 0.816; AP: 0.02, 95% CI: −0.12 to 0.15, *p* = 0.812; SI: 1.12, 95% CI: 0.45–2.80, *p* = 0.804) was observed.

## Discussion

The novelty of our study lies in the evaluation of risk adjusted association between clinically diagnosed DED and sleep disturbances and psychological stress. Furthermore, to our knowledge, this is the first study to explore the potential interaction between these DED risk factors. We found that both sleep disturbances and psychological stress, were significantly associated with an increased risk of DED. However, independently, sleep disturbances and stress elevated DED risk, with no evidence of effect modification on either multiplicative or additive scales. Furthermore, the high prevalence of sleep disturbances, stress, and DED in this sample of higher education students highlights the need for measures to address these public health concerns.

### Why sleep disturbance or stress might be associated with DED?

This study established that a normal sleep pattern is associated with a lower risk of DED. Participants with significant sleep disturbance had higher odds of DED compared with those with normal sleep (adjusted RR 1.11, 95% CI 1.03–1.20). This finding agrees with a systematic review by Gu et al., who found a similar bidirectional association between poor sleep quality and DED. They contend that “sleep deprivation or poor sleep quality may lead to the onset or exacerbation of the symptoms of DED.” [14] The exact mechanism underlying this association remains unclear. However, some studies have offered insights. Lee et al. asserted that impaired sleep quality increases the osmolarity of secreted tears, altering tear homeostasis, which manifests clinically as reduced TBUT [35]. Another school of thought believes that sleep disturbance and deprivation affect the parasympathetic system of the autonomic nervous system, which is responsible for tear secretion in the tear gland. Such disruption affects tear secretion and can lead to DED [36]. Similarly, high psychological stress was associated with the risk of DED (adjusted RR = 1.06, 95% CI: 1.01–1.11; *p* = 0.015), consistent with previous studies, which reported that psychological stress is associated with DED [9,17,37]. The mechanism underlying this association is unclear; however, previous studies have postulated that stress increases systemic inflammatory responses by inducing the production of inflammatory markers, thereby increasing the risk of developing diseases, including DED [17,37].

### What is the implication of these findings? (No effect modification by stress)

Sleep disturbances and stress exhibit a bidirectional relationship: stress can disrupt sleep through anxiety and hyperarousal, while insufficient sleep may heighten physiological and psychological stress responses, creating a self-perpetuating cycle [38]. Hence, we hypothesized that stress might modify the association between sleep disturbances and the risk of DED, given their interrelated nature. In this study, the results from the joint-effect estimates showed the highest risk of DED in students with both poor sleep and high stress (RR 1.18, 95% CI 1.10–1.27, *p* < 0.0001), even though no evidence of effect modification by stress on the association between sleep disturbances and DED risk was found. The absence of evidence for interaction is not necessarily evidence of no interaction as the current study may be underpowered to statistically detect interaction. Hence, the no significant interaction may need to be interpreted with caution.

Notably, sleep disturbances were far more prevalent (75.9%, n = 306) than high stress (21.3%, n = 86) in our participants, aligning with studies of university populations globally [39–41]. These findings underscore that sleep quality could be a critical modifiable risk factor for DED in academic settings, independent of stress. However, the impact of stress cannot be overlooked. To mitigate these risks, evidence-based strategies—such as flexible scheduling, peer support systems, and sleep hygiene programs, stress management strategies including exercising—should be prioritized and encouraged in student health initiatives [35,36,39,40].

### High burden of DED as a public health problem

Results from this study indicate a high prevalence of self-reported DED among the study subjects. The prevalence of symptomatic DED, using the OSDI in this study, was 82.1%. Of this number, 89 (22.1%) participants had mild DED symptoms, 10 (2.5%) had moderate DED symptoms, and 232 (57.5%) reported experiencing severe DED. This result, although higher, is comparable to those from Jordan (72.6%) [17], Poland (57.1%) [6], and Ethiopia. (49.4%) [42] In Ghana, a similar study found the prevalence of symptomatic DED to be 44.3% [9] The higher prevalence of symptomatic DED among university students has been largely attributed to the use of visual display units, reliance on over-the-counter medications, humid areas, and air-conditioned rooms [6,8,9,14]. Following the inclusion of the TBUT, corneal staining and other signs of DED, a somewhat reduced, albeit still higher, prevalence of 77.92% was found. This agrees with the TFOS DEWS II report that “in the absence of objective DED tests, patients will usually overestimate their symptoms of DED” [2]. The results of this study are also comparable to those of clinical testing for DED (using TBUT) among university students in Indonesia (62.7%) [38] and in South Africa (81%) [43]. On the contrary, lower prevalences were reported in Nigeria (28.2%) and Jordan (19.8%) [44,45]. The significantly lower prevalence in these studies may be due to the methods used to clinically diagnose DED. Unlike the current study, the studies in Nigeria and Jordan used anesthetized TBUT. Published prevalence studies among university students, especially in Ghana, have largely relied on symptom questionnaires (OSDI, SPEED, and DEQ-5) rather than on TFOS DEWS II-style multimodal diagnostic criteria.

### Study limitations

This study identified a notably high prevalence of DED among university students. Although several potential confounders—including screen time, alcohol consumption, smoking, and other lifestyle factors—were addressed through eligibility criteria, residual confounding, such as reading duration, outdoor time, caffeine intake, medication use, systemic allergy, mental health symptoms, ocular allergy, humidity exposure, and sleep medication, cannot be entirely excluded. This is inherent to the cross-sectional design and the reliance on self-reported measures, which may be subject to recall and reporting bias. The timing of data collection may also have influenced the findings, as the study was conducted shortly after the COVID‑19 pandemic, a period marked by increased reliance on visual display units (VDUs) for academic activities. While prolonged VDU use (>3 hours/day) was an exclusion criterion, the threshold at which screen exposure has minimal impact on DED remains uncertain, and lower levels of exposure may still have contributed to symptoms. Moreover, the cross-sectional design precludes causal inference and limits assessment of temporal relationships. Future longitudinal studies using objective exposure measures and a larger study population are warranted.

## Conclusion

Sleep disturbance and stress were individually significant risk factors for DED in this student population, but their combined effect did not exceed the sum of their individual associations. Promoting sleep hygiene and stress-management interventions may represent effective strategies for reducing the burden of DED among university students.
